# *Reports on Cities, Towns, and Districts*.—Cardiff

**Published:** 1858-01

**Authors:** 


### SANITARY AND SOCIAL SCIENCE.

### REPORTS ON CITIES, TOWNS, & DISTRICTS.

### SANITARY STATE OF CARDIFF.

The North district of Cardiff comprises that portion of the town which extends from Crockherbtown to Castle Street; it includes all streets opening into this line, being bounded on the south by Quay Street, Church Street, continued on through Ebenezer Street to Charles Street. It is upon a higher level than the other portion of the town. It is inhabited principally by gentry, professional men, and respectable tradesmen. It contains but few courts, these being in good condition, both pitched and paved.

The East district embraces Union Street, East Street, and all the adjacent streets lying between these and the upper part of the Canal, bounded on the north by a portion of that district, and on the south by Bute Terrace and Whitmore Lane, as far as Lewis Street, but does not include that lane. Its inhabitants are a few tradesmen, respectable mechanics, and labourers. Some of the streets—as Stanley Street, Love Lane, and Mary Ann Street—containing Irish lodging-houses. All the streets are pitched and paved; the district has very few courts.

The West district comprises the streets between the Canal, Lewis Street, and the river Taff. In this district, St. Mary Street is inhabited by respectable tradesmen and others. The district has many courts in a bad sanitary condition, as Landore Court, Mill Lane Court, Evans's Court, and others. They are occupied by labourers and indigent Irish, who live in common lodging-houses.

The South district embraces Bute Street, and all the streets in Bute Town; is inhabited by respectable tradesmen, a better class of labourers, and, near the dock, by agents and others connected with the shipping. Nearly the whole of these streets are unpitched, very inefficiently drained, and, being situate on a very low level, usually contain stagnant water and much mud, mixed with refuse vegetable matter. The area behind most of the houses occupied by labourers, being a large proportion, is frequently flooded with fœtid water and cesspool soakings, the alluvial clay, on which this district is built, preventing the water from percolating.

The Newtown district is that portion of the town on the eastern side of the Taff Vale Railway. It has a low level; the streets have recently been pitched; the houses for the most part are occupied as Irish lodging-houses, and are seriously overcrowded.

The drainage of the town, except in the South district, is in rapid progress towards completion; is already made available in removing the surface water from the Newtown district, and has exercised a very beneficial influence on the health of the inhabitants of that district.

The water supply of the town is now good and ample. The Water Works Company supply about 2,700 houses. The water is obtained from Ely, is good for consumption, and, giving only five degrees of hardness, is equally applicable for household purposes. Their scale of charges enables, in many instances where it is desirable, its supply to be enforced, and has been a great boon to occupiers of small tenements.

The meteorological peculiarities of 1855 were as follows:—The winter and spring quarters were unusually cold, the mean temperature being most weeks below the average of previous years, with the exception of a few days in April, but it soon became cold again, and continued so until the end of May. The prevailing winds were N.N.E. In June, the weather was remarkable for sudden changes from heat to cold. In July, the temperature was high; and warm weather continued through August, September, and the early part of October; November was cold and gloomy; the temperature was below the average in the early part of December, but rose the latter end of the month. The fall of rain throughout the year was below the average.

The population of Cardiff is now computed to exceed 25,000, and is supposed to increase yearly at the rate of eight per cent., being the proportionate rate of increase for the ten years previous to the last census, and which increase, we have reason to infer, has been since maintained. The difference therefore between the increase by births, 458, and the actual increase, 2,000, on the population, is made up by the immense yearly immigration.

Assuming the population of Cardiff now to exceed 25,000, and the number of deaths for the year 1655 being 641, the rate of mortality has been 25.64 per thousand, or rather more than 2½ per cent. The rate of mortality of all England is slightly above 2 per cent.; but this includes rural and town districts, and does not therefore contrast fairly with Cardiff, entirely a town district.

Perhaps the most unfavourable aspect under which the mortality of Cardiff, during 1855, can be viewed, is when we consider the rate of infant mortality. Thus, the number of children who died under five years during this period has been 306, or 47.73 on the total number of deaths of all ages.

The greatest evil to which I have to direct attention on the present occasion, is the overcrowded condition of the houses of the poor, especially in the Newtown district. This arises from the disproportion between the inhabitants and the house accommodation; the landlord taking advantage of the great demand by putting on a rent entailing the necessity of the tenant of the house subletting the rooms, the tenant of the room subletting the beds.

A practical illustration of the great advantages that have been derived from this constant sanitary supervision exercised over the districts, formerly the constant habitats of fever and infectious diseases, will be understood by a comparison of their former and present condition. In the year 1849, an inquiry into the sanitary condition of Cardiff was instituted for the first time, and evidence was given as to the state of Stanley-street, Landore-court, and other localities. The subjoined extracts are made from the Poor Law Medical Relief Rook :—

| Locality. | Typhus Fever. 1846-47. Cases. | Typhus Fever. 1847-48. Cases. | Dysentery. 1847-48. Cases. |
|---|---|---|---|
| Stanley Street | 75 | 23 | 13 |
| Landore Court | 48 | 24 | 8 |
| Mary Ann Street | 32 | 4 | 2 |
| Kenton's Court | 14 | 14 | 5 |

In 1849, epidemic cholera prevailed in this town, and caused extensive mortality in the four localities, namely, in—

| | | |
|---|---|---|
| Stanley Street | 19 | deaths. |
| Landore Court | 8 | " |
| Mary Ann Street | 12 | " |
| Kenton's Court | 13 | " |

After this year, the local authorities instituted a system of supervision, and great attention was paid to these and other localities considered the infected districts. In 1854, cholera again broke out in this town. The benefits of this supervision appear by inspection of the Register Book, showing the mortality in the streets just enumerated; while the following is the comparative effect at the two periods :—

| | 1849. | 1854. |
|---|---|---|
| Stanley Street | 19 | 1 |
| Landore Court | 8 | 2 |
| Mary Ann Street | 12 | 2 |
| Kenton's Court | 13 | 0 |

These localities being inhabited as common lodging-houses, admitted of the application of sanitary supervision. But there were certain other localities not coming under this denomination, to which circumstance it could not be enforced, where the epidemic prevailed fatally on the two occasions, in the following proportions :—

| | 1840. | | 1854. |
|---|---|---|---|
| Millicent Street.............. | 18 | ...... | 16 |
| Bridge Street .............. | 5 | ...... | 5 |
| Great Frederick Street ...... | 4 | ...... | 3 |

This exhibits in a marked degree, the beneficial effect of sanitary supervision when epidemics unfortunately prevail.

During the last two years, not a single death from fever has been registered in Stanley Street, Landore Court, Mary Ann Street, or Kenton's Court; while a reference to the Medical Relief Book, since 1849, rarely shews a case of fever in these streets, the same book proving, that before this period, Stanley Street and Landore Court were rarely ever without a fever. The houses are now cleanly and frequently white-limed, ventilation is enforced, and the atmosphere of the rooms is no longer of that sickening and offensive character which they heretofore presented in so marked a degree.

The means necessary to improve the sanitary condition of the town, which I have more particularly to recommend to notice during the coming year, will be—to reduce the number of inmates in houses when overcrowded—to enforce frequent cleansing and white-liming—to compel owners or occupiers of house property to adopt means for removing accumulations of stagnant water and impurities in the back areas—to enforce bye-laws in reference to the practice of throwing vegetables and other matter into the unfinished streets—and to compel the pitching of courts and alleys, the latter of which produced strong evidence of its salutary effect during the prevalence of the cholera epidemic of 1854. (From a report by H. J. Paine, Esq., M.R.C.S., &c., Officer of Health.)

---

